# Arrow to the Chest

**DOI:** 10.5811/cpcem.2019.9.43991

**Published:** 2019-10-21

**Authors:** Sherab Wangdi, Shankar LeVine, Melanie Watts

**Affiliations:** * Khesar Gyalpo University of Medical Sciences of Bhutan, Department of Emergency Medicine, Thimphu, Bhutan; † Jigme Dorji Wangchuck National Referral Hospital, Department of Emergency Medicine, Thimphu, Bhutan

## Abstract

A 33-year-old male was brought to the emergency department after a penetrating arrow injury to the chest. Initial evaluation revealed the arrow was penetrating the sternum, lung, and aortic arch. Because the patient was in a remote area, timely transfer to a specialized center for definitive operative repair was delayed approximately 24 hours. Treatment was focused on minimizing risk of hemorrhage with tight blood pressure control, while tube thoracostomy was deferred to avoid a change in intrathoracic pressure. The left-sided hemothorax was monitored with serial point-of-care ultrasounds. Ultimately he was successfully transferred and underwent successful surgical intervention.

## CASE PRESENTATION

**Image 1:**
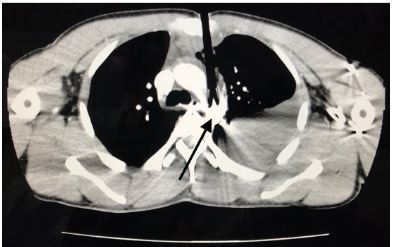
The path of the arrow can be seen, initially penetrating the sternum and anterior lung and ultimately lodging in the aortic arch (arrow).

**Image 2:**
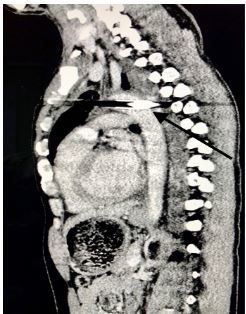
The black arrow reveals the depth of the arrow extending through the aortic arch.

A 33-year-old male was brought to the emergency department following an archery accident. The accident occurred when the patient was competing in an archery tournament and an arrow shot from a compound bow was released from approximately 140 meters away, piercing his chest. Upon initial examination, he was conscious with the arrow piercing his chest at the level of the upper third of the sternum; an estimated 12 centimeters of the arrow was implanted in the chest. He was hemodynamically stable and maintaining his oxygenation. Initial point-of-care ultrasound (POCUS) revealed a left-sided hemothorax without pneumothorax or hemopericardium. An emergent chest computed tomography (CT) revealed the arrow penetrating through the sternum, mediastinum, and passing through the aortic arch (Images 1 and 2) with a moderate left-sided hemothorax. Due to lack of cardiothoracic or trauma surgeons in the country, preparations were made for transfer of the patient for definitive repair.

## DISCUSSION

Traumatic injuries to the thoracic aorta carry a high mortality rate.^1^ Although transesophageal echocardiography was previously considered first line for assessing transthoracic aortic injuries, now its role is limited to unstable patients as CT angiography is the investigational modality of choice.^2^ Hemorrhage control and emergency operative therapy are the mainstay of treatment.^3^ When emergent surgical intervention is not possible optimizing conditions for hemorrhage control is key.

**CPC-EM Capsule**

What do we already know about this clinical entity?

*Traumatic injuries of the thoracic aorta carry a high mortality rate. Computed tomography angiography is the imaging modality of choice. Hemorrhage control is key*.

What is the major impact of the image(s)?

*This unusual image of a penetrating aortic injury with the object in place is illustrative of a situation requiring careful consideration for medical management*.

How might this improve emergency medicine practice?

*This case demonstrates a pathway to optimize conditions for hemorrhage control in resource-poor areas where a delay to definitive surgical treatment is inevitable*.
